# Andes Hantavirus Variant in Rodents, Southern Amazon Basin, Peru

**DOI:** 10.3201/eid2002.131418

**Published:** 2014-02

**Authors:** Hugo Razuri, Rafal Tokarz, Bruno M. Ghersi, Gabriela Salmon-Mulanovich, M. Claudia Guezala, Christian Albujar, A. Patricia Mendoza, Yeny O. Tinoco, Christopher Cruz, Maria Silva, Alicia Vasquez, Víctor Pacheco, Ute Ströher, Lisa Wiggleton Guerrero, Deborah Cannon, Stuart T. Nichol, David L. Hirschberg, W. Ian Lipkin, Daniel G. Bausch, Joel M. Montgomery

**Affiliations:** United States Naval Medical Research Unit No. 6, Lima, Peru (H. Razuri, B.M. Ghersi, G. Salmon-Mulanovich, M.C. Guezala, C. Albujar, A.P. Mendoza, Y.O. Tinoco, C. Cruz, M. Silva, D.G. Bausch, J.M. Montgomery);; Columbia University, New York, New York, USA (R. Tokarz, D.L. Hirschberg, W.I. Lipkin);; Universidad Nacional Mayor de San Marcos, Lima (A. Vasquez, V. Pacheco);; Centers for Disease Control and Prevention, Atlanta, Georgia, USA (U. Ströher, L. Wiggleton Guerrero, D. Cannon, S.T. Nichol);; Tulane School of Public Health and Tropical Medicine, New Orleans, Louisiana, USA (D.G. Bausch)

**Keywords:** hantavirus, Peru, bunyaviruses, epidemiology, zoonoses, viruses, rodents, Neacomys spinosus, mice, Amazon Basin

## Abstract

We investigated hantaviruses in rodents in the southern Amazon Basin of Peru and identified an Andes virus variant from *Neacomys spinosus* mice. This finding extends the known range of this virus in South America and the range of recognized hantaviruses in Peru. Further studies of the epizoology of hantaviruses in this region are warranted.

Hantaviruses are enveloped, tripartite, single-stranded, negative-sense RNA viruses belonging to the genus *Hantavirus*, family *Bunyaviridae*. More than 15 hantaviruses have been recognized in the Americas, most in South America (*1*). Hantaviruses are maintained in rodents and shrews, usually with a tight pairing between the specific virus and host species. On the American continents, hantaviruses can evoke a severe acute disease known as hantavirus pulmonary syndrome (HPS), which carries typical case-fatality rates of 35%–40%, depending on the particular virus (*1*)*.* Hantavirus host–reservoir pairs continue to be discovered, and details of hantavirus epidemiology and the risk for transmission of hantaviruses to humans continue to emerge.

Data on hantaviruses in Peru are sparse and confined to the Loreto Region in the northern Amazon Basin (Figure 1), where a 1996 study of rodents showed a 20% prevalence of IgG to hantaviruses and the identification of a Rio Mamoré–like hantavirus from *Oligoryzomys microtis* rodents (*2*)*.* The IgG prevalence in humans in Loreto tested during 2007–2010 was low (1.7%) (*3*)*.* Nevertheless, 4 cases (3 fatal) of human hantavirus infection were reported in 2011 in this region, 2 from Rio Mamoré and 2 from Seoul virus (*4*,*5*)*.* To expand the knowledge base on hantaviruses in Peru, we conducted an investigation of rodents in a previously unexplored area of Peru’s southern Amazon Basin.

**Figure 1:**
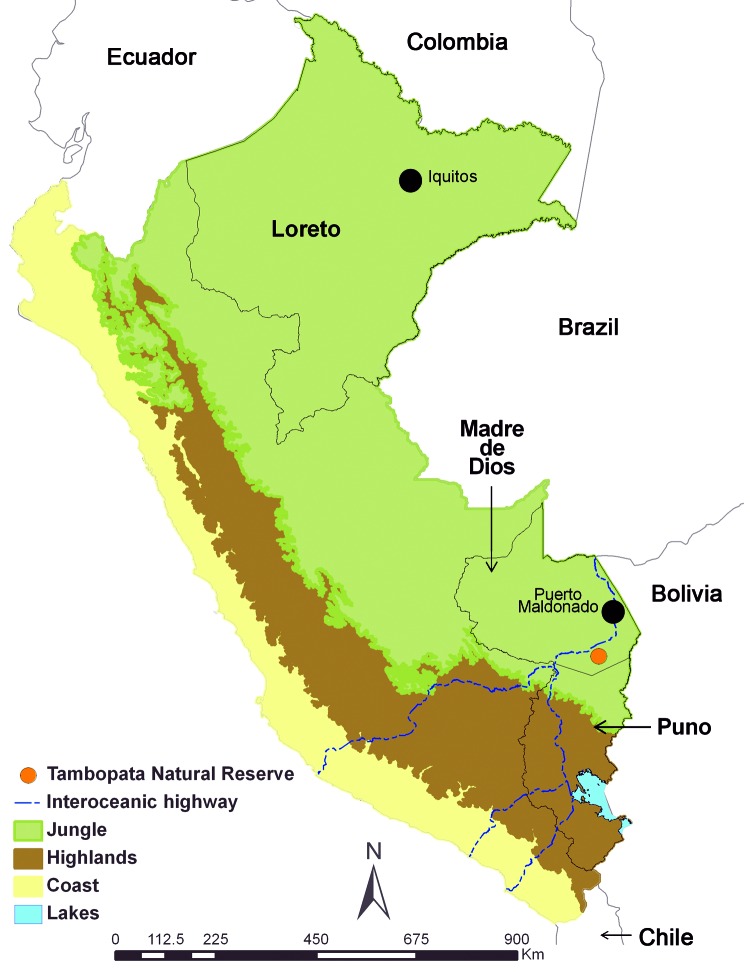
Regions of Peru, indicating areas of previous hantavirus study (Loreto [*2*]) and the study of hantaviruses described in this article (Madre de Dios and Puno). Capital cities of the Loreto and Madre de Dios Regions are indicated by black dots.

## The Study

In 2003, Peru began construction on an interoceanic highway through the southern Amazon Basin in the Peruvian administrative regions of Madre de Dios and Puno; the highway is intended to link Amazon sites with Pacific ports (Figure 1). During October 2009–October 2010, we collected small mammals at 6 sites in the region of the highway construction as part of a study exploring the effects of this construction on the region’s fauna and flora, including the risk for rodent-borne disease. This study was approved by the US Naval Medical Research Unit No. 6 Institutional Animal Care and Use Committee, and collection permits were obtained from the Peruvian Ministry of Agriculture Office of Forestry and Wildlife. Animals were collected in 6 trapping sessions by using live traps (H.B. Sherman Traps, Tallahassee, FL, USA; Tomahawk Live Trap, Hazelhurst, WI, USA) and standard methods (*6*)*.* We also collected animals at a relatively undisturbed site near the Tambopata National Reserve (Figure 1). Animals were anesthetized and euthanized before morphometric measurements were taken and necropsies performed. Samples of blood, heart, lung, spleen, liver, and kidney were collected (*7*); tissues were stored in liquid nitrogen for transport to the laboratory. A section of liver was also preserved in 95% ethanol for molecular identification of rodent species. Rodent carcasses were fixed in 10% formalin and preserved in 70% ethanol as permanent voucher specimens.

Rodent blood was tested by ELISA for IgG to New World hantaviruses at the Centers for Disease Control and Prevention Special Pathogens Branch in Atlanta, Georgia, USA, by using nucleocapsid antigen derived from Sin Nombre virus, which is broadly cross-reactive among New World hantaviruses (*6*)*.* Nested reverse transcription PCR (RT-PCR) was performed by Naval Medical Research Unit No. 6 on homogenized lung samples from IgG-positive rodents. PCR targetED a 434-bp region of the hantavirus small (S) segment and 296- and 242-bp regions of the medium (M) segment G1 and G2 regions, respectively (*8*)*.* Ion Torrent Personal Genome Machine (Life Technologies, Carlsbad, CA, USA) high-throughput sequencing and additional consensus RT-PCR on ELISA-positive rodents were performed at Columbia University in New York, NY, USA. Rodent species was determined on the basis of morphometric assessment by experienced mammalogists at the San Marcos University Natural History Museum in Lima, Peru; identification of IgG-positive animals was confirmed by DNA extraction from the liver followed by PCR amplification and sequencing of the cytochrome *b* gene (*9*)*.*

During a total of 15,145 trap-nights, 362 rodents (trapping success rate 2.4%) belonging to 14 species were captured. The most frequently trapped rodents were 155 (42.8%) *Oligoryzomys microtis* mice, 49 (13.5%) *Necromys lenguarum* mice, 41 (11.3%) *Hylaeamys* spp. rodents, 35 (9.7%) *Euryoryzomys nitidus* rats, and 34 (9.4%) *Neacomys spinosus* mice. Six (1.7%) rodents had positive results for hantavirus IgG: 2 *N. spinosus* mice (both adult males), 2 *N. lenguarum* mice (1 adult and 1 juvenile, both females), 1 *H. yunganus* rat (juvenile female), and 1 *E. nitidus* rat (adult male). IgG-positive rodents were trapped at 5 times from 3 sites at distances 43–145 km from Puerto Maldonado, the capital city of Madre de Dios. 

**Figure 2:**
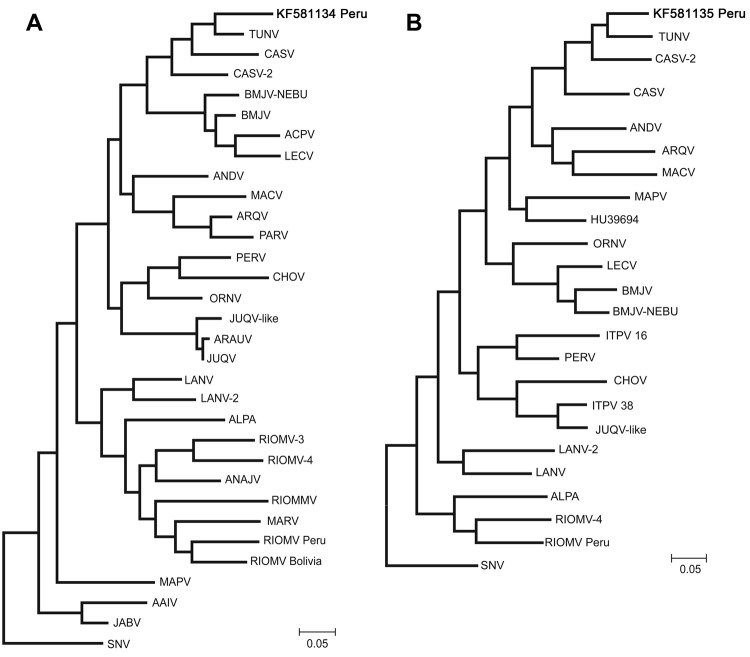
Maximum likelihood phylogenetic trees of the small (S; panel A) and medium (M; panel B) segments of the hantavirus identified in this study in Peru (boldface) compared with segments of hantaviruses from throughout South America. Trees were generated by using MEGA5.2 software (www.megasoftware.net) with 2,000 bootstrap replicates. All viruses are shown relative to North American Sin Nombre virus (SNV; GenBank accession nos. L25784, L25783 [for M segment]). Hu39694 (AF028023 [M]) is also shown. TUNV, Tunari virus (GenBank accession nos. JF750417 [S], JF750420 [M]); CASV, Castelo dos Sonhos virus (AF307324, HQ719471 [S]; JX443698, JX443699 [M]); BMJV-NEBU, Bermejo-Neembucu virus (DQ345763 [S], AY515603 [M]); BMJV, Bermejo virus (AF482713 [S], AF028025 [M]); ACPV, Andes Central Plata virus (EU564715 [S]); LECV, Lechiguanas virus (AF482714 [S], AF028022 [M]); ANDV, Andes virus (AF004660 [S], AF324901 [M]); MACV, Maciel virus (AF482716 [S], AF028027 [M]); ARQV, Araraquara virus (AF307325 [S], AY970821 [M]); PARV, Paranoa virus (EU643620 [S]); PERV, Pergamino (AF482717 [S], AF028028 [M]); CHOV, Choclo virus (DQ285046 [S], DQ285047 [M]); ORNV, Oran virus (AF482715 [S], AF028024 [M]); JUQV-like, Juquitiba-like (GU213197 [S], AY963900 [M]); ARAUV, Araucaria (AY740628 [S]); JUQV, Juquitiba (EF492472 [S]); LANV, Laguna Negra virus (AF005727 [S], FJ816031 [S], AF005728 [M], JX443703 [M]); ALPA, Alto Paraguay virus (DQ345762 [S], AY515602 [M]); RIOMV, Rio Mamore virus (FJ532244, U52136, JX443667, JX443679 [S]; FJ608550 [M], JX443701 [M]); ANAJV, Anajatuba virus (JX443690 [S]); RIOMMV, Rio Mearim virus (DQ451828 [S]); MARV, Maripa virus (GQ179973 [S]); MAPV, Maporal virus (FJ008979 [S], AY363179 [M]); AAIV, Ape Aime Itapua virus (GU205340 [S]); JABV, Jabora virus (JN232079 [S]); and ITPV, Itapua virus (16, AY515606 [M]; 38, AY515604 [M]). Scale bars indicate nucleotide substitutions per site.

Results of RT-PCR testing were positive for 2 of 6 IgG-positive rodents, the 2 *N. spinosus* mice, which were trapped on consecutive days at the same site, 80 km from Puerto Maldonado. However, a hantavirus sequence was identified in only 1 of these rodents by both consensus RT-PCR and high-throughput sequencing. Assembly of hantavirus reads obtained by high-throughput sequencing resulted in the generation of 1,068 bp of the S, 1,673 bp of the M, and 572 bp of the large (L) segments for the virus. Phylogenetic analysis showed the virus to be an Andes virus clade variant most similar to viruses within the Castelo dos Sonhos (CASV) group, which consists of CASV and CASV-2, found in Brazil, and Tunari virus (TUNV), found in Bolivia (Figure 2) (*10*)*.* The nucleotide sequences of the S and M segments were 91% and 90%, respectively, identical to TUNV (98% and 97% aa identity, respectively). The L segment sequence was most similar to CASV-2 (85% nt and 98% aa identity). No TUNV L segment sequence data are available for comparison. The S, M, and L nucleotide sequences for the virus we identified were deposited in GenBank under accession nos. KF581134, KF581135, and KF581136, respectively.

## Conclusions

Our data confirm the presence of an Andes virus variant similar to TUNV and CASV in the southern Amazon Basin of Peru; this finding extends the known range of this virus in South America and the range of recognized hantaviruses in Peru. HPS has been associated with CASV in Brazil (*11*,*12*) and with TUNV in Bolivia (*13*). However, no cases have been reported in the southern Amazon Basin of Peru, nor is formal surveillance for the disease conducted in the area, and no human antibody prevalence studies have been conducted. The lack of reported human cases could mean that the frequency of infected rodents, or of human contact with them, is low; that human infection occurs but is asymptomatic or too mild to be recognized; or that HPS is misdiagnosed by clinicians who are unfamiliar with the condition and lack access to diagnostic testing. In some areas of South America, including Bolivia, Paraguay, Argentina, and Chile, a high prevalence of hantavirus IgG in humans has been reported, but reports of cases of HPS have been infrequent (*14*)*.*

*Oligoryzomys* spp. rodents appear to be the principal reservoirs for most Andes viruses, including CASV (*12*,*15*); no reservoir has been associated with TUNV. Our data suggest that *N. spinosus* mice may be the reservoir for the Andes virus variant found in Madre de Dios and Puno, although spillover infection from an alternate reservoir cannot be excluded. If these mice are the reservoir for this virus, the human population at risk for hantavirus infection by transmission from *N. spinosus* mice could be large, because the range of this species encompasses considerable portions of Brazil, Colombia, Ecuador, Peru, and Bolivia. *N. spinosus* mice have not been associated with other hantaviruses. Further investigation of the epizoology of hantaviruses and possible risks to humans in the southern Amazon Basin of Peru is warranted.
